# Deciphering the Salt Tolerance Mechanisms of the Endophytic Plant Growth-Promoting Bacterium *Pantoea* sp. EEL5: Integration of Genomic, Transcriptomic, and Biochemical Analyses

**DOI:** 10.3390/biology15010045

**Published:** 2025-12-26

**Authors:** Zonghao Yue, Mengyu Ni, Nan Wang, Jingfang Miao, Ziyi Han, Cong Hou, Jieyu Li, Yanjuan Chen, Zhongke Sun, Keshi Ma

**Affiliations:** 1 College of Life Sciences and Agronomy, Zhoukou Normal University, Zhoukou 466001, China; yzh@zknu.edu.cn (Z.Y.); nimengyu123@163.com (M.N.); gaolenghanbaobao@163.com (N.W.); wsxderfcvg@163.com (J.M.); shipinhanziyi@163.com (Z.H.); houcongyyqx@163.com (C.H.); lijieyusp@163.com (J.L.); 2 Field Observation and Research Station of Green Agriculture in Dancheng County, Zhoukou 466001, China; 3 School of Mechanical and Electrical Engineering, Zhoukou Normal University, Zhoukou 466001, China; 20172033@zknu.edu.cn; 4 College of Biological Engineering, Henan University of Technology, Zhengzhou 450001, China

**Keywords:** *Pantoea*, ST-PGPB, Na^+^ extrusion, antioxidant defense, compatible solutes, energy metabolism

## Abstract

Soil salinization poses a significant threat to global agricultural productivity. Salt-tolerant plant growth-promoting bacteria (ST-PGPB) have shown great potential in enhancing crop resilience under saline stress, yet the molecular basis of their intrinsic tolerance remains incompletely understood. To address this, we employed an integrated genomic, transcriptomic, and biochemical approach to investigate the salt tolerance strategies of *Pantoea* sp. EEL5, an endophytic ST-PGPB isolated from *Elytrigia elongata*. The results demonstrated that EEL5 exhibited remarkable salt tolerance and efficiently removed Na^+^ via extracellular adsorption and intracellular accumulation. Genomic analysis identified key genes responsible for Na^+^ efflux, betaine synthesis and transport, and typical plant growth-promoting traits. Under salt stress, transcriptomic profiling revealed a marked upregulation of genes involved in Na^+^ extrusion, antioxidant enzymes, betaine biosynthesis and transport, arginine and proline catabolism, TCA cycle, and electron transport chain, concomitant with a downregulation of genes governing energy-intensive flagellar assembly and chemotaxis. These coordinated responses facilitated Na^+^ exclusion, enhanced antioxidant capacity, accumulated compatible solutes (betaine, glutamate, and GABA), increased energy production, and conserved energy via motility reduction, collectively conferring salt tolerance in EEL5. Our findings elucidate the multi-level salt adaptation mechanisms of EEL5 and provide a genetic foundation for a comprehensive understanding of ST-PGPB.

## Simple Summary

Soil salinization is a major threat to crops worldwide. Although some salt-tolerant growth-promoting bacteria (ST-PGPB) can confer crops tolerance to high salt, their own resilience mechanisms are not fully known. This study investigated the salt tolerance mechanisms of an endophytic ST-PGPB *Pantoea* sp. EEL5 isolated from *Elytrigia elongata*. Under salt stress, this bacterium actively removed Na^+^ from its cells, enhanced its antioxidant defenses, and accumulated protective molecules like betaine, glutamate, and GABA. It also increased energy production while conserving resources by reducing its mobility. These coordinated changes allowed it to thrive under high-salt conditions. The findings reveal the multi-layered strategy behind ST-PGPB’s salt tolerance, offering a molecular basis for developing more effective microbial products to improve crop resilience in salty fields.

## 1. Introduction

Soil salinization poses a major global challenge to agricultural sustainability. According to the FAO, more than 1.38 billion hectares of land worldwide are affected by salinity, accounting for approximately 10.7% of the global land area [1]. Alarmingly, another 1.04 billion hectares face potential risks of salinization. Although plants possess inherent mechanisms to cope with salt stress, these are often insufficient under severe or chronic salinity. High soil salinity primarily disrupts ionic homeostasis and causes osmotic stress, which in turn damages cellular integrity, triggers a burst of reactive oxygen species (ROS), and inhibits photosynthesis, ultimately impeding crop growth and reducing yield [2,3]. It is estimated that salt stress leads to an annual relative crop yield loss of up to 0.5% globally [1]. Given that the global population is projected to reach 9.7 billion by mid-21st century, developing effective strategies to enhance crop resilience in saline soils has become imperative for safeguarding agricultural output and meeting future food requirements.

Microorganisms play an essential role in maintaining soil health and establishing plant-microbe symbiotic systems. Hence, beneficial microbes, particularly salt-tolerant plant growth-promoting bacteria (ST-PGPB), has emerged in recent years as a highly promising and sustainable strategy to enhance crop performance in saline soils [4,5]. These ST-PGPB typically possess various plant growth promoting (PGP) traits and can survive well in saline environments through employing adaptive mechanisms such as Na^+^ extrusion, compatible solute accumulation, biofilm formation, exopolysaccharide secretion, and the regulation of salt-responsive genes [6]. Most importantly, they can enhance crop resistance to soil salinization through producing phytohormones, promoting photosynthesis, maintaining ion homeostasis, accumulating osmolytes, activating antioxidant defenses, and regulating gene expression [7]. Many ST-PGPB, such as *Bacillus*, *Azotobacter*, *Enterobacter*, *Pseudomonas*, and *Burkholderia*, have been successfully used as bioinoculants to enhance crop growth and yield in saline soils [8]. However, reports on ST-PGPB from other genera remain limited.

A representative example of these understudied genera is *Pantoea*. Bacteria in this genus can function either as pathogens or as beneficial agents, depending on the specific strain and host plant [9]. Recently, as several species within this genus have demonstrated significant potential as ST-PGPB in enhancing plant stress resistance [10,11,12,13], strains from the *Pantoea* genus have attracted increasing scientific interest. Notably, the intrinsic salt tolerance mechanisms of *Pantoea* species are still poorly understood. To bridge this knowledge gap, we focused on a promising ST-PGPB strain, *Pantoea* sp. EEL5, which was previously isolated from the *Elytrigia elongata*. This strain exhibits robust salt tolerance (up to 120 g/L NaCl) and multiple PGP traits, and has shown efficacy in enhancing wheat resilience under combined stress [14]. Its superior salt tolerance compared to other reported Pantoea strains makes it an ideal model for mechanistic studies.

Therefore, this study employed an integrated approach combining genomic, transcriptomic, and biochemical analyses to elucidate the salt tolerance mechanisms of strain EEL5. Our findings are expected to elucidate novel salt adaptation mechanisms in *Pantoea*-derived ST-PGPB and inform the development of more effective microbial agents.

## 2. Materials and Methods

### 2.1. Growth Curve of EEL5 and Removal Efficiencies of Na^+^ Under Different NaCl Stress

Strain EEL5 was inoculated into 20 mL sterilized Luria–Bertani (LB) broth containing 1–10% (*w*/*v*) NaCl (initial OD_600_ = 0.01). These cultures were incubated at 30 °C and 180 rpm. Bacterial growth was monitored by measuring the OD_600_ every 4 h using a microplate reader (Molecular Devices, Sunnyvale, CA, USA). After 24 h of cultivation, 5 mL aliquots of the culture were centrifuged at 8000× *g* for 5 min to collect the supernatants. The supernatants were then filtered through a 0.22 μm sterile membrane, and the Na^+^ concentration was determined using a A3AFG flame atomic absorption spectrophotometer (FAAS) (Purkinje General Instrument Co., Ltd., Beijing, China). The Na^+^ removal efficiency was calculated as follows: Removal efficiencies (%) = (C_0_ − C_24_)/C_0_ × 100, where C_0_ and C_24_ represent the Na^+^ concentrations (g/L) at 0 and 24 h, respectively.

### 2.2. Extracellular Adsorption and Intracellular Bioaccumulation of Na^+^ by EEL5

Strain EEL5 was inoculated into sterilized LB broth containing 6% NaCl (initial OD_600_ = 0.01). After 12 h of cultivation at 30 °C and 180 rpm, 50 mL of the culture was collected and centrifuged at 8000× *g* for 10 min. The supernatant was discarded, and the bacterial pellets were washed twice with sterile water and subsequently resuspended in 15 mL of 4 mM EDTA solution (pH 8.0). The suspensions were shaken at 220 rpm for 25 min and then centrifuged again at 8000× *g* for 10 min. The resulting supernatants, containing the extracellularly adsorbed Na^+^, were collected for analysis. The cell pellets, representing the intracellularly accumulated Na^+^, were dried at 60 °C for 24 h, weighed, and digested using a mixture of 68% HNO_3_ and 30% H_2_O_2_ (4:1, *v*/*v*) in a digestion instrument. The digested samples were diluted to a final volume of 25 mL with deionized water. Both the EDTA-eluted supernatants and the digested solutions were filtered through a 0.22 μm membrane, and their Na^+^ concentrations were quantified using a A3AFG FAAS. The amounts of extracellularly adsorbed (Ea) and intracellularly accumulated (Ia) Na^+^ were calculated using the following equations: Ea (mg/g) = (Ce × Ve)/m/1000 and Ia (mg/g) = (Ci × Vi)/m/1000, where Ce (mg/L) and Ve (mL) are the Na^+^ concentration in the EDTA eluent and the volume of the eluent, respectively; Ci (mg/L) and Vi (mL) are the Na^+^ concentration in the bacterial digestate and the volume of the digestate, respectively; m (g) was the dry weight of the bacterial cells.

### 2.3. Scanning Electron Microscopy and Energy Dispersive X-Ray Spectroscopy (SEM-EDS) Analysis of EEL5

For SEM-EDS analysis, strain EEL5 was cultured in LB broth containing 6% NaCl for 12 h. Bacterial cells were harvested by centrifugation at 8000× *g* for 10 min at 4 °C, washed twice with PBS buffer (0.01 M, pH 7.4), and subsequently fixed. Fixation was performed sequentially with 2.5% glutaraldehyde for 2 h and 1% osmic acid for another 2 h at room temperature. The fixed cells were then dehydrated through a graded ethanol series (30% to 100%) and critically point-dried. The dried samples were sputter-coated with a thin gold layer using an ion sputter coater (Hitachi High-Tech, Tokyo, Japan). Morphological observation and elemental analysis were conducted using a SU8100 SEM equipped with an Ultim Max 100 EDS detector (Oxford Instruments, Abingdon, Oxfordshire, UK).

### 2.4. Draft Genome Sequencing and Bioinformatic Analyses of EEL5

Genomic DNA was extracted from EEL5 in the logarithmic growth phase using a commercial bacterial DNA extraction kit (Majorbio, Shanghai, China). After quantification, the DNA was sheared into approximately 400 bp fragments. Sequencing libraries were constructed with the NEXTflex Rapid DNA-Seq kit (PerkinElmer, Waltham, MA, USA) and paired-end sequenced on an Illumina HiSeq X platform (San Diego, CA, USA) by Majorbio (Shanghai, China). Raw reads were quality-controlled using Fastp (v0.20.0) to obtain high-quality clean reads, which were then assembled into scaffolds using SOAP de novo (v2.04) and GapCloser (v1.12). Protein-coding sequences (CDSs), rRNA, tRNA and ncRNA predictions were performed using Prodigal (v2.6.3), Barrnap (v0.9), tRNA-scan-SE (v2.0.12) and Infernal (v1.1.5), respectively. CDSs were functionally annotated by aligning against the NR, Swiss-Prot, Pfam, GO, COG, KEGG and CAZY databases (e-value < 10^−5^). Secondary metabolite biosynthetic gene clusters (BGCs) were predicted using antiSMASH (v7.0.0).

### 2.5. Transcriptome Sequencing of EEL5 Under NaCl Stress

After a 12 h cultivation in LB broth supplemented with 6% NaCl, EEL5 cells were collected and snap-frozen in liquid nitrogen. Total RNA was extracted using a cetyltrimethylammonium bromide (CTAB) method. Following quality and integrity assessment, rRNA was removed from the total RNA using the RiboCop rRNA Depletion Kit for Mixed Bacterial Samples (Lexogen, Vienna, Austria) to enrich mRNA. Purified mRNA was randomly sheared into 200 bp fragments and used to construct transcriptome libraries with the Stranded mRNA Prep Ligation (Illumina, San Diego, CA, USA). Libraries were sequenced on the Illumina HiSeq X-PLUS platform (San Diego, CA, USA) by Majorbio. Raw reads were processed using Fastp (v0.20.0) to generate clean reads, which were then aligned to the EEL5 draft genome using Bowtie2 (v2.5.4). Gene expression levels were quantified with RSEM (v1.3.3) and reported in transcripts per million (TPM). Differentially expressed genes (DEGs) were identified using DESeq2 (v1.42.0) under the criteria of adjusted *p* value < 0.01 and |log2(fold change)| ≥ 1.0. Functional enrichment analysis of DEGs was conducted through Gene Ontology (GO) and Kyoto Encyclopedia of Genes and Genomes (KEGG) databases using Goatools (v1.4.4) and KOBAS (v2.0), respectively.

### 2.6. Validation of Quantitative Real-Time PCR (qPCR)

To validate the transcriptomic results, 20 DEGs were selected for qPCR analysis (primer sequences are provided in Supplementary Table S1). Total RNA samples from the transcriptome study were reverse-transcribed into cDNA using HiScript II Q RT SuperMix (Vazyme, Nanjing, China). qPCR was performed on a CFX96 Real-Time PCR System (Bio-Rad, Hercules, CA, USA) with ChamQ SYBR qPCR Master Mix (Vazyme, Nanjing, China). The housekeeping gene *rpoB* was used as the internal control for normalization, as it showed stable expression under our experimental conditions according to the transcriptome data. Relative gene expression levels were calculated using the 2^−ΔΔCt^ method.

### 2.7. Biochemical Measurements

After being cultured in 6% NaCl for 12 h, EEL5 cells were harvested, subjected to 15 min of sonication on an ice bath, and then centrifuged to collect the supernatant for biochemical analysis. The contents of superoxide anion (O_2_^−^), malondialdehyde (MDA), adenosine triphosphate (ATP), betaine, Glutamate (Glu), and γ-aminobutyric acid (GABA) were measured using commercial kits provided by Boxbio (Beijing, China). The hydrogen peroxide (H_2_O_2_) content was quantified with a commercial kit purchased from Elabscience (Wuhan, China). All kits were used according to the manufacturers’ protocols.

### 2.8. Effects of Exogenous Betaine, Glu, and GABA on the Growth of EEL5 Strain Under Salt Stress

To evaluate whether the increase in compatible solutes could enhance the salt resistance of EEL5, the strain was inoculated into 5 mL sterilized LB broth containing 6% NaCl (initial OD_600_ = 0.01). The culture medium was supplemented with 150 mg/L betaine, 1.0 mM Glu, or 2.0 mM GABA, with an unsupplemented medium as the control. All cultures were incubated at 30 °C and 180 rpm for 12 h. Bacterial growth was assessed by measuring the OD_600_ using a microplate reader (Molecular Devices, Sunnyvale, CA, USA).

### 2.9. Statistical Analysis

Statistical analyses were performed using SPSS statistics software 19.0 (IBM, Armonk, NY, USA). Data are presented as mean ± SD from at least three biological replicates. For comparisons between two groups, an unpaired Student’s *t*-test was applied. For comparisons among four groups, a one-way analysis of variance (ANOVA) with Tukey’s test was conducted. *p* value <0.05 were considered statistically significant.

## 3. Results

### 3.1. EEL5 Exhibits High Removal Capabilities of Na^+^

**Figure 1:**
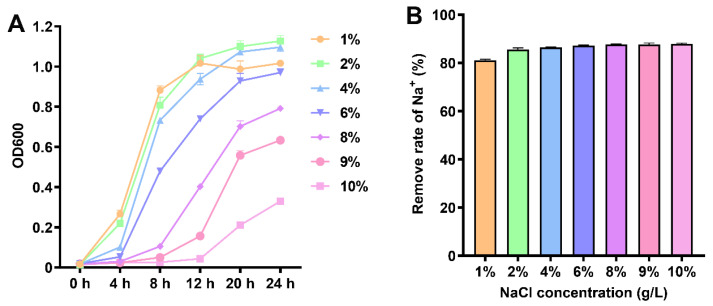
Growth dynamics and Na+ removal of strain EEL5 under NaCl stress. (**A**) Growth curve of EEL5 under 1–10% NaCl stress; (**B**) Na^+^ removal efficiency of EEL5 under 1–10% NaCl stress.

As shown in Figure 1A, strain EEL5 demonstrated robust growth under NaCl stress, with no significant inhibition at concentrations up to 6% after 24 h culture. Further increase in NaCl concentration led to a dose-dependent growth inhibition. Nevertheless, the bacterial biomass at 10% NaCl remained at approximately one-third of that measured at 1% NaCl. Notably, despite this growth inhibition, EEL5 maintained a consistently high Na^+^ removal efficiency, exceeding 80% across the entire NaCl concentration range tested (1–10%) (Figure 1B).

### 3.2. EEL5 Removes Na^+^ Through Extracellular Adsorption and Intracellular Accumulation

**Figure 2:**
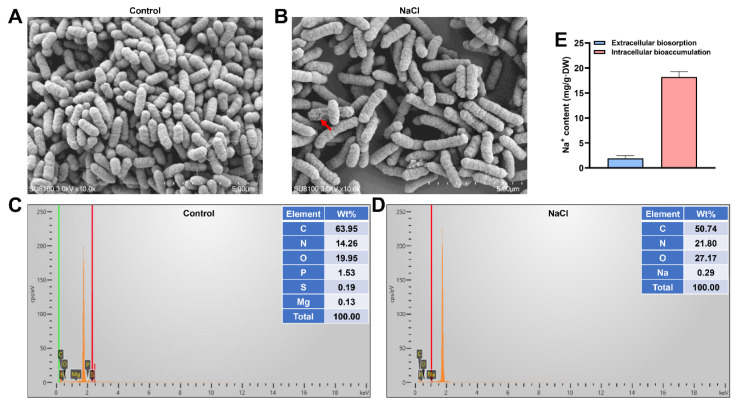
SEM-EDS analysis and quantification of extracellularly adsorbed and intracellularly accumulated Na^+^ in strain EEL5 exposed to 6% NaCl for 12 h. (**A**,**B**) SEM images of bacterial cells in the Control and NaCl groups, scale bar: 5 μm, Red arrow: cell with surface shrinkage; (**C**,**D**) EDS spectrograms of bacterial cells in the Control and NaCl groups, different colored lines represent the characteristic X-ray peaks corresponding to different elements; (**E**) Na^+^ contents of extracellular adsorption and intracellular accumulation under NaCl stress.

SEM imaging revealed a typical short-rod morphology in control cells (Figure 2A), whereas NaCl stress induced widespread cellular elongation, with a minority of cells exhibiting noticeable surface shrinkage (Figure 2B). EDS analysis confirmed the presence of Na^+^ on the surface of NaCl-stressed cells, providing direct evidence of extracellular adsorption (Figure 2D). Quantitative measurements of Na^+^ distribution showed that EEL5 effectively removed Na^+^ via both extracellular adsorption and intracellular accumulation (Figure 2E). Notably, intracellular accumulation played a dominant role in Na+ removal by EEL5.

### 3.3. Genomic Features and Functional Potential of Strain EEL5

**Figure 3:**
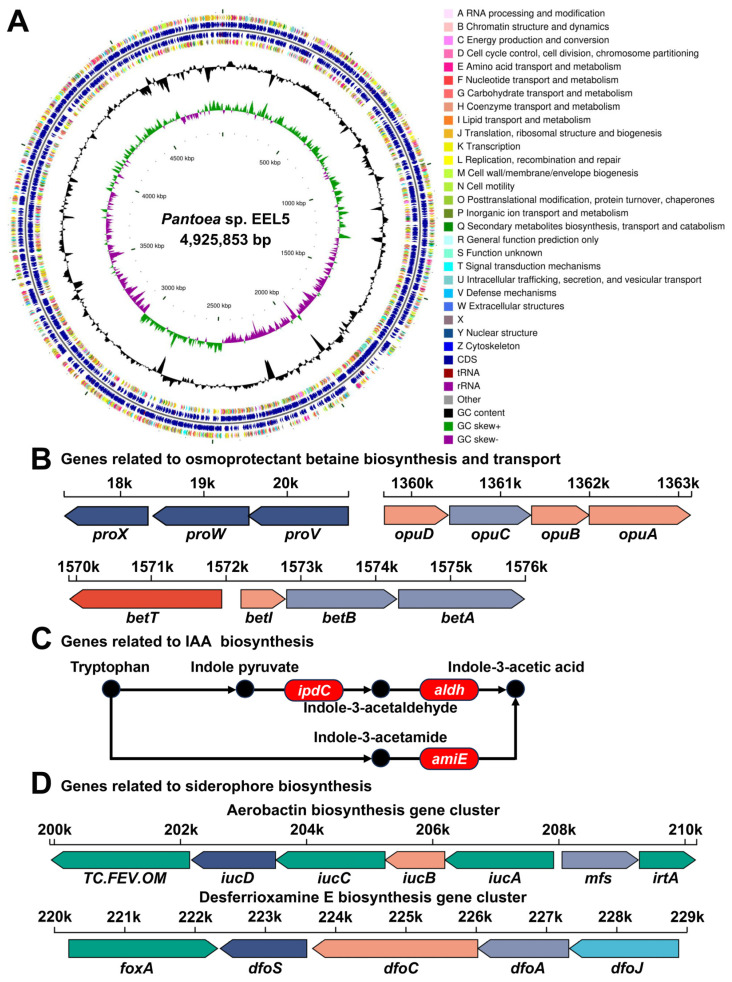
Draft genome of strain EEL5 and identified key genes. (**A**) Genomic circle map drawn using CGView; (**B**) Genes related to betaine biosynthesis and transport; (**C**) Genes related to IAA biosynthesis; (**D**) Genes related to siderophore production.

Draft genome sequencing of strain EEL5 yielded 9.50 million clean reads. De novo assembly produced a 4.93 Mb genome distributed across 21 scaffolds, with an N50 of 578.8 kb and a largest scaffold of 2.45 Mb (Figure 3A, Table S2). Genome annotation predicted 4417 CDSs, 69 tRNAs, 6 rRNAs and 104 ncRNA (Table S2). Functional analysis identified key genes related to Na^+^ efflux, such as *nhaA*, *nhaK*, and *nha1* encoding Na^+^/H^+^ antiporters and *yrbG* encoding Na^+^/Ca^2+^ antiporter (Table S3). The genome also harbored complete gene clusters for the synthesis and uptake of the osmoprotectant betaine, including the *betABIT* and *opuABCD* systems, as well as the *proVWX* genes (Figure 3B, Table S4). Furthermore, we identified multiple genes associated with PGP traits, encompassing indole-3-acetic acid (IAA) biosynthesis, siderophore production, and phosphorus solubilization (Figure 3C,D, Table S4).

### 3.4. Transcriptomic Analysis of Strain EEL5 Under NaCl Stress

**Figure 4:**
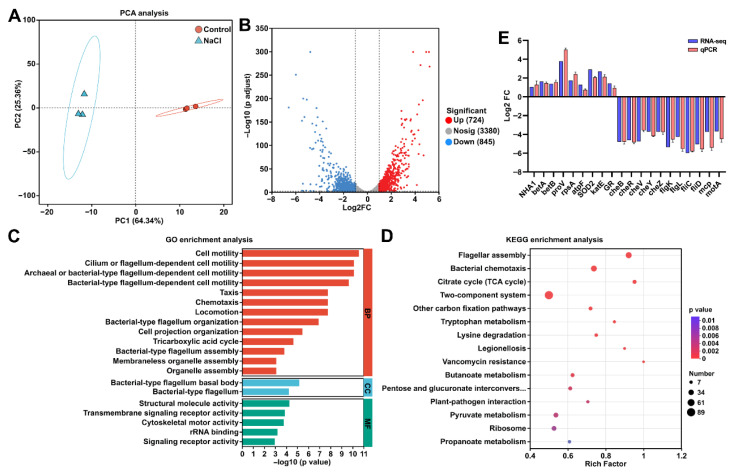
Transcriptomic analysis of strain EEL5 under NaCl stress. (**A**) PCA; (**B**) Volcano plot of differentially expressed genes (DEGs). (**C**) GO enrichment analysis of DEGs. The top 20 enriched GO terms were displayed. (**D**) KEGG pathway enrichment analysis of DEGs. (**E**) qPCR validation of transcriptome data.

Principal component analysis (PCA) revealed a clear separation between the Control and NaCl-stressed samples, indicating distinct global transcriptomic profiles in response to salt stress (Figure 4A). Transcriptome sequencing generated a total of 150.61 million raw reads. After stringent quality control, 122.90 million high-quality clean reads (Q30 > 94.40%) were obtained, of which 97.57% to 99.55% were successfully mapped to the EEL5 draft genome (Tables S5 and S6). Comparative analysis between the Control and NaCl-treated groups identified 1569 DEGs, comprising 724 upregulated and 845 downregulated genes (Figure 4B). GO enrichment analysis revealed that these DEGs were significantly enriched in 136 terms. The most significantly affected terms were primarily associated with cell motility, chemotaxis, flagellar assembly, and the tricarboxylic acid (TCA) cycle (Figure 4C). Additionally, notable enrichment was observed in terms related to transmembrane transport, as well as the biosynthesis and catabolism of key metabolites such as betaine, arginine, and glutamine-family amino acids (Table S7). KEGG pathway enrichment analysis showed that these DEGs were significantly enriched in 14 pathways. Among these, “Flagellar assembly”, “Bacterial chemotaxis”, and “TCA cycle” were identified as the most prominently altered pathways under NaCl stress (Figure 4D). In addition, qPCR analysis of selected genes confirmed expression trends consistent with the transcriptome data, supporting the reliability of the transcriptome results (Figure 4E).

### 3.5. EEL5 Activates Na^+^ Efflux and the Biosynthesis of Compatible Solutes Under NaCl Stress

**Figure 5:**
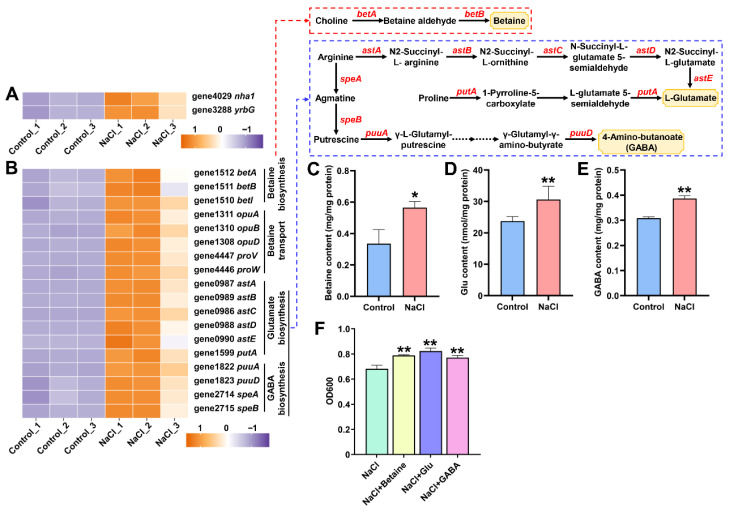
The expression levels of genes related to compatible solutes and the contents of compatible solutes in EEL5 cells under NaCl stress. (**A**) DEGs related to Na+ efflux. (**B**) DEGs involved in betaine biosynthesis and transport, and arginine and proline metabolism. (**C**) Betaine content. (**D**) Glu content. (**E**) GABA content. (**F**) The growth of EEL5 with or without betaine, Glu or GABA under NaCl stress. * *p* < 0.05, ** *p* < 0.01.

Under NaCl stress, the expression of Na^+^ efflux genes (*nha1* and *yrbG*) and key genes involved in betaine biosynthesis and transport (*betA*, *betB*, *opuA*, *opuB*, *opuD*, *proV*, and *proW*) was significantly upregulated (Figure 5A,B). In parallel, transcriptional activation was observed in genes associated with arginine metabolism (*astA*, *astB*, *astC*, *astD*, *astE*, *speA*, *speB*, *puuA*, and *puuD*) and proline metabolism (*putA*) (Figure 5B). Consistently, the intracellular contents of betaine, Glu, and GABA were markedly elevated under NaCl stress (Figure 5C,E). Furthermore, supplementation with these compatible solutes significantly enhanced the growth of EEL5 under salt stress, indicating their functional role in improving osmotic tolerance (Figure 5F).

### 3.6. EEL5 Actives Antioxidant Enzyme Defense System to Protect Cells from Oxidative Stress

**Figure 6:**
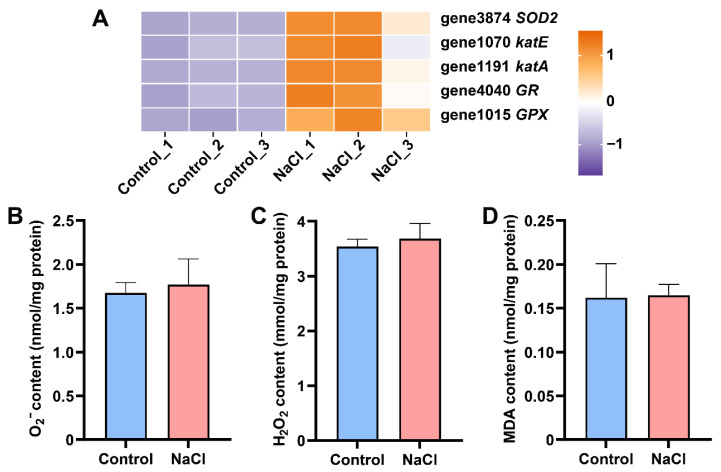
The expression levels of antioxidant enzyme genes and ROS levels of EEL5 under NaCl stress. (**A**) DEGs involved in antioxidant enzymes. (**B**) O_2_^−^ content. (**C**) H_2_O_2_ content. (**D**) MDA content.

Under NaCl stress, EEL5 significantly upregulated the expression of key antioxidant enzyme genes, including those encoding superoxide dismutase (SOD2), catalase (CAT), glutathione peroxidase (GPX), and glutathione reductase (GR) (Figure 6A). Interestingly, there is no significant difference in O_2_^−^, H_2_O_2_ and MDA contents between the Control and NaCl groups (Figure 6B,D).

### 3.7. NaCl Stress Reprograms Energy Metabolism by Repressing Motility and Enhancing Respiration

**Figure 7:**
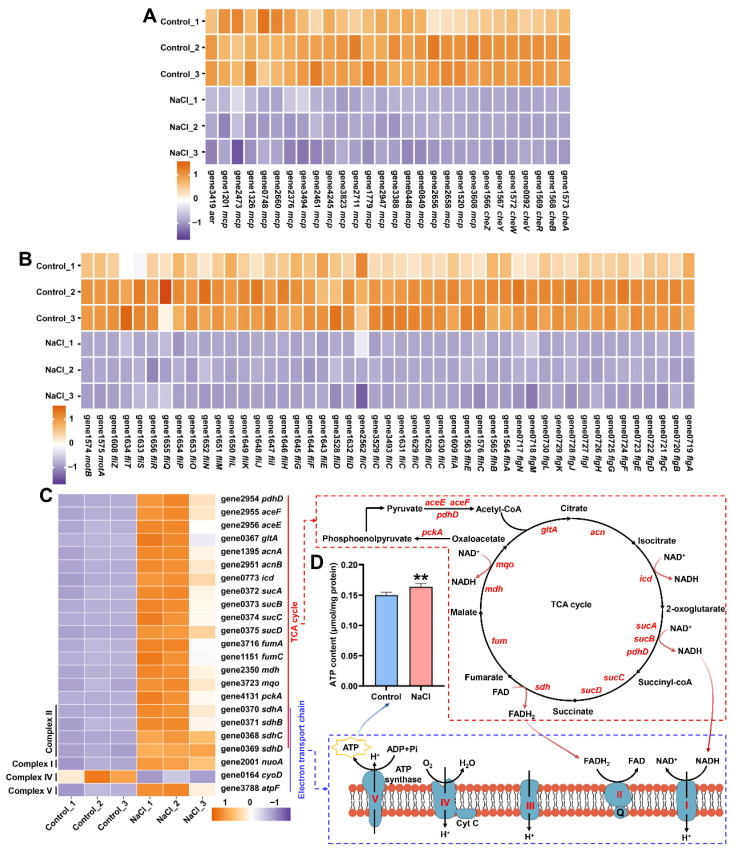
The expression levels of genes related to flagellar assembly (**A**), chemotaxis (**B**), and TCA cycle/electron transport chain (**C**) in EEL5 cells under NaCl stress and the content of intracellular ATP (**D**). Complex I (I): NADH dehydrogenase; Complex II (II): Succinate dehydrogenase; Complex III (III): Cytochrome bc1 complex; Complex IV (IV): Cytochrome c oxidase; Complex V (V): ATP synthase; CoQ10 (Q): Coenzyme Q10; Cyt C: Cytochrome c. ** *p* < 0.01.

Under NaCl stress, a notable downregulation was detected for all DEGs associated with flagellar assembly and chemotaxis (Figure 7A,B). Conversely, DEGs involved in the TCA cycle and those encoding Complexes I, II, and V of the electron transport chain exhibited a marked upregulation (Figure 7C). Correspondingly, a significant increase in intracellular ATP content was observed in EEL5 subjected to NaCl stress (Figure 7D).

## 4. Discussion

*Pantoea* sp. EEL5 is a strain of ST-PGPB that we previously isolated [8]. In this study, EEL5 growth was unimpaired at 6% NaCl and could still reach one-third of that under 1% NaCl even at 10% NaCl, again confirming the high salt tolerance of EEL5. This level of tolerance was notably higher than that of other reported *Pantoea* strains [10,15]. Furthermore, EEL5 maintained high Na^+^ removal efficiency across a wide range of NaCl concentrations, suggesting that this strain has inherent regulatory mechanisms that enable it to survive and exhibit superior Na^+^ removal efficiency even under high-salt conditions. To understand how strain EEL5 remove Na^+^, the SEM-EDS analysis was performed. SEM observations revealed the cell elongation and surface shrinkage at 6% NaCl, indicating the morphological response to cellular stress. Such NaCl-induced morphological changes have also been reported in other strains [16,17,18]. Further EDS elemental analysis confirmed the binding of Na+ to the EEL5 cell surface, demonstrating the occurrence of extracellular adsorption. Moreover, the intracellular Na^+^ content was found to be significantly higher than the extracellularly adsorbed Na^+^, indicating that Na^+^ removal by EEL5 primarily relies on intracellular accumulation. This efficient accumulation suggested that EEL5 possessed sophisticated ion homeostasis and osmotic balance systems, as supported by genomic sequencing, which identified key Na^+^ efflux genes along with genes involved in the biosynthesis and transport of the compatible solute betaine. Moreover, the genome harbored a suite of genes responsible for synthesizing IAA, secreting siderophores, and solubilizing phosphate, which explained the PGP traits of EEL5 documented in our prior research [14]. Collectively, these genomic findings provided a genetic basis for the salt tolerance and the multifaceted PGP capabilities of strain EEL5.

To elucidate the molecular mechanisms underlying salt tolerance in EEL5, we conducted transcriptomic analysis of cells exposed to NaCl. Transcriptomic data revealed that NaCl stress triggered extensive transcriptional reprogramming in EEL5. Among the DEGs, those encoding Na^+^ efflux systems, particularly *nha1* and *yrbG*, were significantly upregulated. NHA1, a plasma membrane-localized Na^+^/H^+^ antiporter, facilitates Na^+^ efflux and has been shown to enhance salt tolerance in Saccharomyces cerevisiae when overexpressed [19,20]. Similarly, YrbG functions as a Na^+^/Ca^2+^ antiporter, exporting Na^+^ in exchange for Ca^2+^ [21]. The upregulation of both antiporters in EEL5 demonstrated that it initiated Na^+^ extrusion to maintain intracellular Na^+^ homeostasis.

It has been reported that numerous salt-tolerant microorganisms adapt to high-salinity by accumulating compatible solutes to balance osmotic pressure and stabilize protein structure and function [22,23]. In this study, NaCl stress significantly upregulated DEGs related to compatible solutes, including those for betaine biosynthesis and transport, arginine metabolism, and proline metabolism. In bacteria, *betA* and *betB* encode enzymes that convert choline to betaine, while *opuABCD* and *proVWX* encode transporters for betaine transport [24,25,26]. Arginine is converted to Glu through the arginine succinyltransferase (AST) pathway involving *astABCDE* genes, and to GABA via the arginine decarboxylase (ADC) pathway involving *speAB* and *puuABCD* genes [27]. Additionally, proline can be converted to Glu by the bifunctional enzyme encoded by *putA* [28]. Therefore, the transcriptional activation of these pathways led to the accumulation of compatible solutes betaine, Glu, and GABA. Previous studies have confirmed that the application of exogenous betaine, Glu, or GABA can promote cell growth under high-salt conditions [29,30]. Similarly, in this study, the supplementation of these three substances significantly improved salt tolerance of L5 strain.

Generally, high-salinity stress causes oxidative damage in cells by inducing excessive accumulation of reactive oxygen species (ROS). In response, microorganisms activate enzymatic antioxidant systems, including superoxide dismutase (SOD), catalase (CAT), and glutathione peroxidase (GPX), to mitigate such damage [31]. In this defense cascade, SOD catalyzes the dismutation of O_2_^−^ into hydrogen peroxide (H_2_O_2_), which is subsequently detoxified to water and oxygen by CAT or GPX [32,33]. Consistent with this general strategy, EEL5 mounted a robust antioxidant response under NaCl stress, as evidenced by the significant upregulation of genes encoding SOD, CAT, GPX, and GR. The efficacy of this transcriptional activation was confirmed by the stable, unstressed cellular levels of O_2_, H_2_O_2_, and MDA, demonstrating that the induced defense system effectively neutralized ROS and protected the cells from oxidative damage.

Microorganisms often inhibit non-essential and energy-consuming physiological processes to cope with environmental stress [34]. In the present study, NaCl stress triggered the significant downregulation of numerous DEGs related to flagellar assembly and chemotaxis in EEL5. The flagellum, the largest self-assembling protein structure in bacteria, serves as the primary motility organelle in most species [35]. Its assembly is a complex and energetically costly process that requires the coordinated expression of dozens of genes [36]. Furthermore, flagellar-driven motility is governed by a sophisticated chemotaxis system composed of methyl-accepting chemotaxis proteins (MCPs) and core signaling components such as *CheA*, *CheW*, *CheY*, *CheB*, *CheZ*, and *CheR* [37]. These components collectively sense environmental cues and transduce signals to the flagellar motor, thereby modulating bacterial movement [38]. Therefore, the observed coordinated suppression of flagellar assembly and chemotaxis implied that EEL5 adopted an energy-saving strategy under high-salinity conditions by reducing motility to reallocate resources toward stress adaptation. Similar observations of reduced motility and chemotaxis have been reported in *Bacillus cereus* and *Pseudomonas stutzeri* under NaCl stress [39,40].

In parallel with the repression of energy-costly processes, transcriptomic analysis revealed a marked upregulation of genes involved in the tricarboxylic acid (TCA) cycle and the electron transport chain (ETC). The TCA cycle, as the central hub of aerobic metabolism, oxidizes acetyl-CoA to generate reducing equivalents (NADH and FADH2), which subsequently deliver electrons to the ETC [41]. This electron transfer drives the establishment of a proton gradient that powers ATP synthase to produce ATP through oxidative phosphorylation [42]. Consistent with this transcriptional rewiring, intracellular ATP levels were significantly elevated under NaCl stress. Taken together, these results demonstrated that EEL5 enhanced its bioenergetic capacity under high salinity, thereby supplying the requisite ATP for energy-demanding salt tolerance mechanisms.

## 5. Conclusions

**Figure 8:**
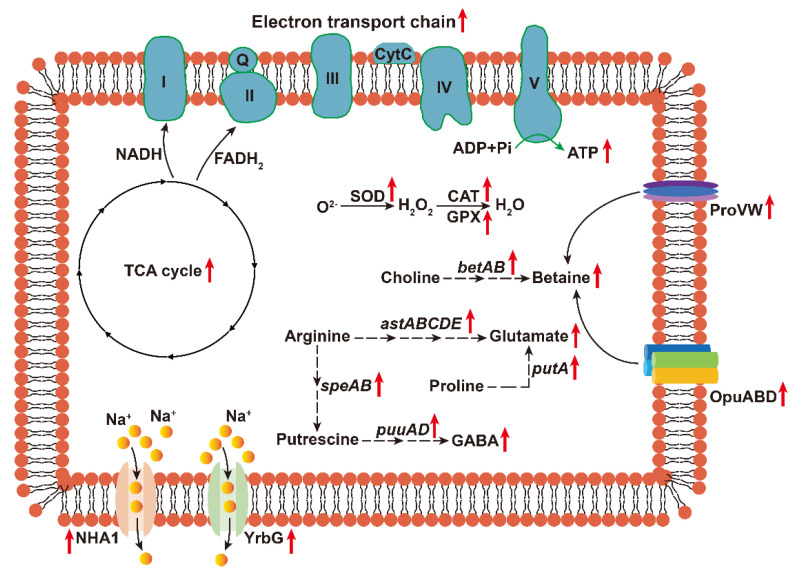
Schematic diagram of the salt tolerance mechanism in EEL5.

In conclusion, our findings unveiled that *Pantoea* sp. EEL5 possessed a remarkable capacity for Na^+^ removal, achieved primarily through extracellular adsorption and intracellular accumulation. The salt tolerance of EEL5 was underpinned by a multifaceted mechanism, which included the enhancement of Na^+^ efflux, accumulation of compatible solutes (e.g., betaine, glutamate, and GABA), activation of an efficient antioxidant defense system, and a metabolic reprogramming characterized by the coordinated enhancement of energy generation alongside the conservation of energy (Figure 8). Our study provided the genetic groundwork and mechanistic understanding crucial for engineering-enhanced ST-PGPB.

## Data Availability

The annotated draft genome sequence of the EEL5 is available at the NCBI GenBank database under the accession number JBNDNJ000000000. The transcriptome data can be found at the GSA database of China National Center for Bioinformation (CNCB) under the accession number CRA032697. Data will be made available on request.

## Supplementary Materials

The following supporting information can be downloaded at: https://www.mdpi.com/article/10.3390/biology15010045/s1, Table S1: The primer sequences for qPCR assay; Table S2: Genome statistical information for *Pantoea* sp. EEL5; Table S3: Genes related to Na^+^ efflux; Table S4: Genes related to betaine and PGP traits; Table S5: Summary of the RNA-seq data; Table S6: The mapped rate of the RNA-seq data; Table S7: GO terms related to transmembrane transport, betaine biosynthesis and transport, arginine and glutamine-family amino acids catabolism.
